# Macrophage Migration Inhibitory Factor (MIF) and Its Homologue D-Dopachrome Tautomerase (DDT) Inversely Correlate with Inflammation in Discoid Lupus Erythematosus

**DOI:** 10.3390/molecules26010184

**Published:** 2021-01-01

**Authors:** Rosario Caltabiano, Rocco De Pasquale, Eliana Piombino, Giorgia Campo, Ferdinando Nicoletti, Eugenio Cavalli, Katia Mangano, Paolo Fagone

**Affiliations:** 1 Department of Medical, Surgical and Advanced Technologies “G.F. Ingrassia”, University of Catania, Via Santa Sofia, 87, 95123 Catania, Italy; rocaltab@unict.it (R.C.); elianapiombino@hotmail.it (E.P.); 2 Department of General Surgery and Medical-Surgical Specialties, University of Catania, 95123 Catania, Italy; r.depasquale@unict.it; 3 Department of Biomedical and Biotechnological Sciences, University of Catania, 95123 Catania, Italy; giorgiacampo.96@gmail.com (G.C.); eugeniocavalli9@hotmail.it (E.C.); kmangano@unict.it (K.M.); paolofagone@yahoo.it (P.F.)

**Keywords:** discoid lupus erythematosus, macrophage migration inhibitory factor, d-dopachrome tautomerase

## Abstract

Discoid Lupus Erythematosus (DLE) is a chronic cutaneous disease of unknown etiology and of immunoinflammatory origin that is characterized by inflammatory plaques and may lead to disfiguring scarring and skin atrophy. Current treatments are limited, with a large proportion of patients either poorly or not responsive, which makes DLE an unmet medical need. Macrophage migration inhibitory factor (MIF) is the prototype of a pleiotropic family of cytokine that also includes the recently discovered homologue D-dopachrome tautomerase (DDT) or MIF2. MIF and DDT/MIF-2 exert several biological properties, primarily, but not exclusively of a proinflammatory nature. MIF and DDT have been suggested to play a key role in the pathogenesis of several autoimmune diseases, such as multiple sclerosis and type 1 diabetes, as well as in the development and progression of certain forms of cancers. In the present study, we have performed an immunohistochemistry analysis for the evaluation of MIF in DLE lesions and normal skin. We found high levels of MIF in the basal layer of the epidermis as well as in the cutaneous appendage (eccrine glands and sebocytes) of normal skin. In DLE lesions, we observed a significant negative correlation between the expression of MIF and the severity of inflammation. In addition, we performed an analysis of MIF and DDT expression levels in the skin of DLE patients in a publicly available microarray dataset. Interestingly, while these in silico data only evidenced a trend toward reduced levels of MIF, they demonstrated a significant pattern of expression and correlation of DDT with inflammatory infiltrates in DLE skins. Overall, our data support a protective role for endogenous MIF and possibly DDT in the regulation of homeostasis and inflammation in the skin and open up novel avenues for the treatment of DLE.

## 1. Introduction

Lupus erythematosus (LE) is an autoimmune disease with apparent multiple etiology, characterized by the presence of activated immune cells and autoantibodies, which leads to diffuse tissue damages. LE may have broad clinical manifestations, include cutaneous lesions. Cutaneous lesions can be observed in absence of systemic signs of the diseases, or in the presence of systemic disease, namely systemic lupus erythematosus (SLE) [1]. The cutaneous forms of LE can be classified as acute cutaneous LE, subacute cutaneous LE, and discoid lupus erythematosus (DLE). 

DLE is characterized by erythematous macules or papules, etelangiectasias, and resolution may lead to scar formation. It has been demonstrated that cytotoxic lymphocytes can destroy the stem cells of hair follicles and cause the scarring of skin DLE lesions.

Unlike patients with SLE, those affected by DLE do not exhibit high titers of anti-Ro antibodies which might indicate the absence of a systemic autoreactivity in DLE. Nonetheless, it is known that patients with DLE are at a risk to develop SLE [1,2], and have a higher incidence of developing squamous cell carcinoma in the scarred areas [3]. Drug treatments of DLE include topical agents (steroids and calcineurin inhibitors) and systemic agents, such as hydroxychloroquine, retinoids, and azathioprine [3]. Early treatment may lead to disease remission, however, ineffective treatment may result in permanent scarring. Involvement of the skin of the fingers and toes could lead to impaired hand function and restrict walking. Additionally, DLE patients are, as stated above, at higher risk to develop SLE [4].

Macrophage Migration Inhibitory Factor (MIF) is a pleiotropic cytokine with upstream regulatory roles in innate and adaptive immunity, which is endowed with multiple proinflammatory properties [5,6,7,8,9,10]. MIF is a highly conserved protein of 12.5 kDa, with evolutionarily ancient homologues in plants, protozoans, nematodes, and invertebrates. The gene in the human genome is located on chromosome 22 (22q11.2), along with at least nine other homologue genes, including the recently identified homologue d-dopachrome tautomerase (d-DT or MIF-2), that shares most, but not all, the biological activities of MIF and may, therefore, represent an endogenous amplificator of the action of MIF [8]. MIF actively participates in multiple stages of the inflammatory response, acting on cells directly and/or potentiating the effects exerted by other stimuli. MIF overcomes the inhibitory effects of glucocorticoids on TNF alpha, IL-1 beta, IL-6, and IL-8 production by LPS-stimulated monocytes in vitro and suppresses the protective effects of steroids against lethal endotoxemia in vivo [8]. MIF also antagonizes glucocorticoid inhibition of T-cell proliferation in vitro, by restoring IL-2 and IFN-gamma production [8,9]. MIF has been found to activate the extracellular signal-regulated kinase 1 (ERK1)/ERK2, members of the family of mitogen-activated protein kinases (MAPKs), upon binding of CD74 and consequent recruitment of CD44 [9]. Additionally, MIF exhibits chemokine-like properties via non-similar binding to the chemokine receptors, CXCR2, CXCR4 and CXCR7. Moreover, following endocytosis, MIF binds the intracellular protein JAB1 (known as JUN-activation domain-binding protein 1), thus inhibiting its downstream pathways, represented by the activation of AP-1 and the degradation of p27Kip1 [9,11,12]. Recent evidence indicates that MIF is centrally implicated in the activation of the inflammasome [13,14]. In agreement with these proinflammatory properties, preclinical and clinical studies by ourselves and others have indicated that MIF is implicated in the pathogenesis of autoimmune diseases, including rheumatoid arthritis (RA), type 1 diabetes, and multiple sclerosis and Guillain-Barré syndrome [5,6,7,8,9,10,15,16]. We have also proposed that upregulated production of MIF may contribute to the immunoinflammatory events occurring in cases of major depressive disorders [17].

More in particular, with regard to the role of MIF in SLE, preclinical studies have shown that negating the action of endogenous MIF ameliorate the course of SLE-like syndrome in mice and that in peripheral blood mononuclear cells, MIF selectively induces the production of the proinflammatory cytokines, IL-6 and TNF-alpha.

However, other studies confute the unique proinflammatory nature of MIF as it has been shown that MIF can stimulate the secretion of both T helper type 1 and 2 (Th1 and Th2) cytokines, as well as interleukin (IL)-17 by lymph node cells, suggesting no single clear role in T cell polarization [18,19]. MIF has also been proposed to function as an immunosuppressive cytokine that contributes to immune privilege, in particular by inhibiting NK cells activity [20]. Recent studies have shown that the interaction of MIF with its receptor CD74 has been associated to PD-L1 expression and IFN-γ production, thus favoring an immunosuppressive environment that may favor immunosuppression and tumor evasion in melanoma [21].

Along this line of research, several studies by ourselves and others have proposed a pathogenetic role of MIF, and possibly DDT, in certain cancers such as melanoma, neuroblastoma, head and neck cancer, and glioblastoma [9,22,23,24,25,26].

These studies have, therefore, evidenced pleiotropic roles of MIF and DDT in the modulation of immune responses and indicate that the ultimate nature of their pro- or anti-inflammatory effects depend on different variables, including local vs. peripheral concentration, type of cells implicated in the immune response, and genetic predisposition. Recently, Benedek and coworkers [6] have demonstrated that sex may also influence the production of MIF and DDT, as these cytokines were found primarily augmented in male patients with MS. However, we could not observe, here or in our previous studies, a sex-biased production of MIF and DDT, in particular, in MS patients and patients with episodes of clinically isolated syndromes of MS [7,15].

To date, little is known about the involvement of MIF and DDT in DLE. In the present study, we have evaluated the expression levels of MIF in skin biopsies of DLE patients and normal controls. The levels of MIF were also correlated to the inflammatory status and to the clinical characteristics of the patients. The data were further enriched in silico, by analyzing a publicly available microarray dataset. The levels of MIF and DDT were determined and correlated to the type of immune cell infiltrations. We found high levels of MIF in the basal layer of epidermis as well as in the cutaneous appendage (eccrine glands and sebocytes) of normal skin. In DLE lesions, we observed a significant negative correlation between the expression of MIF and the severity of inflammation. These data were further corroborated by an analysis of MIF expression levels in a publicly available microarray dataset. Interestingly, while these in silico data only showed a trend toward reduced expression of MIF in the skin of DLE patients, they dismantled a similar pattern of expression and correlation with inflammatory infiltrates of the MIF homologue, DDT. Overall, our data support a protective role for the MIF family of cytokines MIF and its homologue DDT in the regulation of homeostasis and inflammation in the skin and open up novel avenues for the treatment of DLE.

## 2. Results

### 2.1. Immunohistochemistry Analysis of MIF

**Figure 1:**
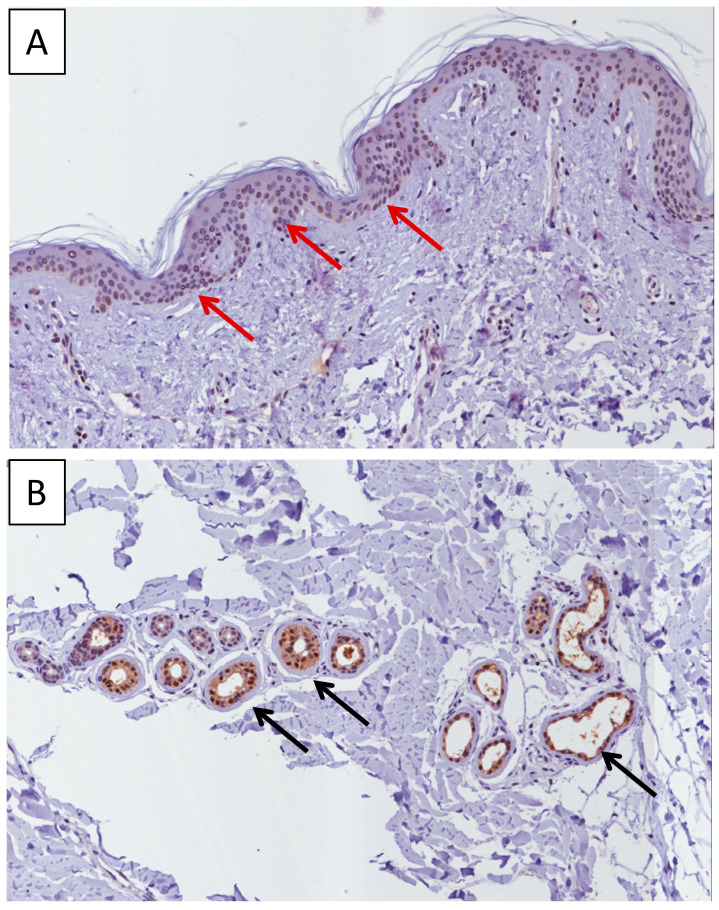
Immunohistochemistry analysis of MIF expression in normal skin biopsies. High levels of MIF can be detected at the basal layer of the epidermis (red arrow) (**A**) and in the cutaneous appendages (eccrine glands and sebocytes) (black arrow) (**B**). Immunohistochemistry-positive staining was defined as the presence of brown chromogen detection within the cytoplasm. Representative microphotographs are shown.

Analysis of the expression of MIF in skin biopsies of healthy donors revealed that high levels of MIF can be detected at the basal layer of the epidermis (Figure 1A) and in the cutaneous appendages (eccrine glands and sebocytes) (Figure 1B).

**Figure 2:**
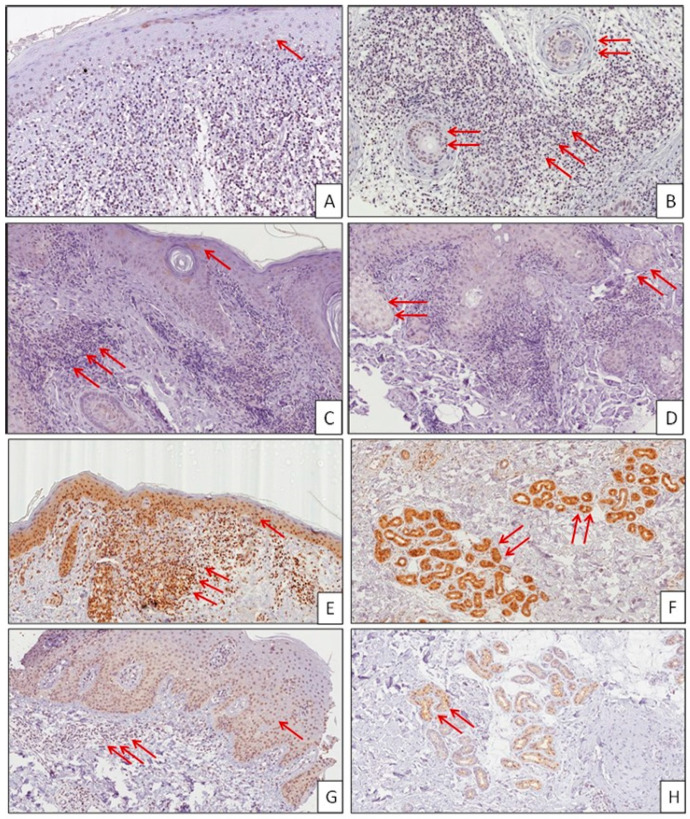
Immunohistochemistry analysis of MIF expression in DLE skin biopsies. Thirty-seven patients with DLE were recruited for this study. Biopsies were performed after a six-month wash-out period from topical treatments. Hematoxylin and eosin staining was used for histopathological evaluation. Representative microphotographs are shown. (**A**–**D**) In the presence of a high inflammatory score (2+; 3+), MIF low expression (0; 1+) was observed in the epidermis (one arrow), appendages (two arrows), and inflammatory infiltrate (three arrows). ((**A**,**B**): patient no. 1; (**C**,**D**): patient no. 2). (**E**–**H**) In the presence of a low inflammatory score (0; 1+), MIF high expression (2+; 3+) was observed in the epidermis (one arrow), appendages (two arrows), and inflammatory infiltrate (three arrows). Immunohistochemistry positive staining was defined as the presence of brown chromogen detection within the cytoplasm. (**E**,**F**): patient no. 3; (**G**,**H**): patient no. 4).

**Figure 3:**
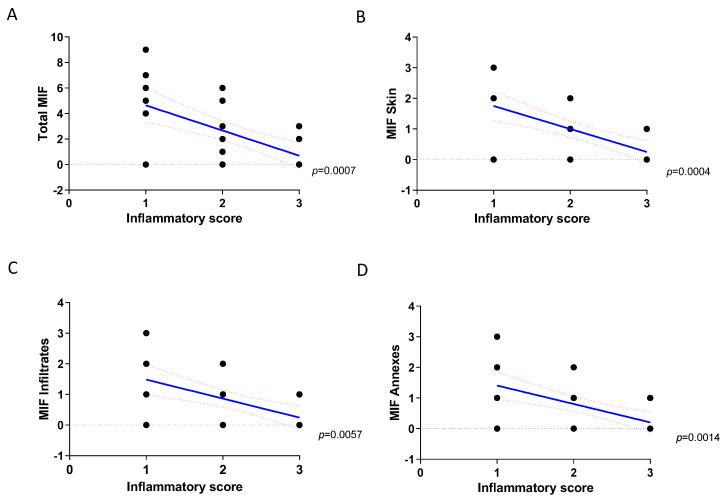
Correlation analysis between MIF expression and inflammatory score in DLE skin biopsies. Thirty-seven patients with DLE were recruited for this study. Biopsies were performed after a six-month wash-out period from topical treatments. Hematoxylin and eosin staining was used for histopathological evaluation. (**A**) Correlation between total MIF expression and inflammatory score. (**B**) Correlation between MIF expresses in the basal layer of the epidermis and the inflammatory score. (**C**) Correlation between MIF expression in the infiltrate and the inflammatory score. (**D**) Correlation between MIF expression in the annexes and the inflammatory score. Correlation analysis was performed using the non-parametric Spearman’s test.

In DLE patients (Table 1), we observed a significant negative correlation between the expression of MIF and the severity of inflammation (*p* < 0.01 by Spearman correlation test) (Figure 2 and Figure 3A). The same negative correlation was also observed when we compared the inflammatory score with the expression of MIF in the basal layer of the epidermis (Figure 3B), in the inflammatory infiltrate (Figure 3C) and in the annexes (Figure 3D), which entailed a strong statistical significance (*p* = 0.0004, *p* = 0.0057 and *p* = 0.0014, respectively).

**Figure 4:**
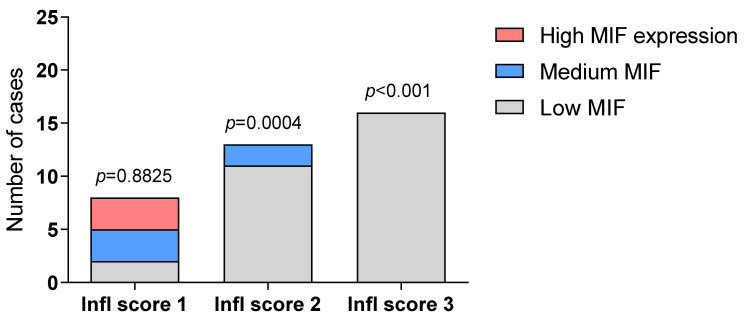
Relative proportions of DLE samples with different expression of MIF and increasing inflammatory score. Thirty-seven patients with DLE were recruited for this study. Biopsies were performed after a six-month wash-out period from topical treatments. Hematoxylin and eosin staining was used for histopathological evaluation. Statistical analysis was performed using the Fisher’s chi-square test. Infl score: Inflammatory score.

Accordingly, the highest proportion of patients expressing low levels of MIF showed a high inflammatory score (2+; 3+) (*p* < 0.001 by the chi-square test) (Figure 4) and high MIF expression was exclusively found in samples with low inflammatory score (Figure 4).

No correlation was found between MIF levels and patient clinical data, including age, sex, BMI, and response to treatment (Table 2).

### 2.2. In Silico Study

**Figure 5:**
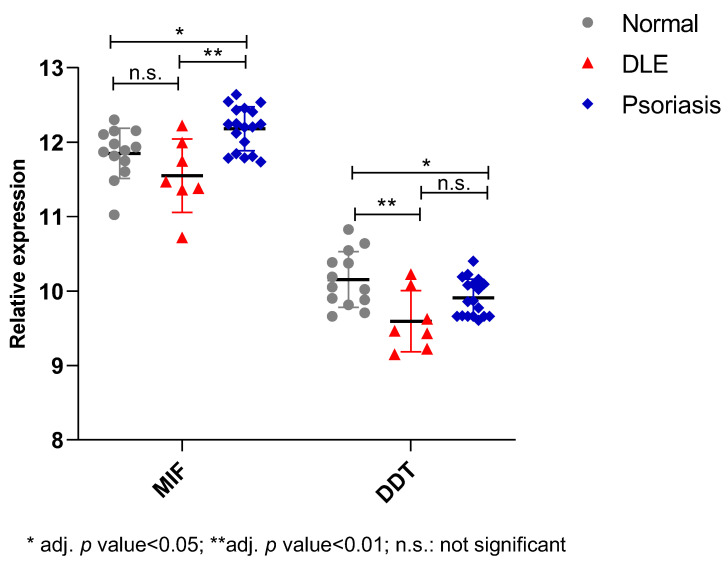
Relative expression analysis of MIF and DDT in DLE samples. Determination of the expression levels of MIF and DDT was performed by interrogating the GSE52471 microarray dataset, retrieved from the Gene Expression Omnibus databank (GEO; https://www.ncbi.nlm.nih.gov/gds). The GSE52471 dataset included whole genome expression profiles of skin biopsies from seven DLE patients, 13 healthy donors, and 18 psoriatic patients. The LIMMA (Linear Models for Microarray Analysis) R package was used for identifying differentially expressed genes. An adjusted *p*-value < 0.05 was considered as the threshold of significance.

In order to confirm the immunohistochemistry analysis, we interrogated the GSE52471 microarray dataset, which included whole genome expression profiles of skin biopsies from seven DLE patients, 13 healthy donors, and 18 psoriatic patients. As shown in Figure 5, a trend of reduced expression levels of MIF (*p* = 0.18) was found in DLE samples, as compared to normal skin samples. Interestingly, in psoriatic lesions used as an additional positive control for immunoinflammatory disease of the skin, MIF levels were, on the contrary, significantly increased as compared to normal skin levels (Figure 5).

As overlapping structural and biological properties are shared between MIF and DDT, we also evaluated DDT levels in the same dataset. As shown in Figure 5, DDT expression profile showed a significant reduction in DLE and psoriatic samples (Figure 5). This data, along with the immunohistochemistry analysis, suggests that the reduced levels of MIF may largely depend on a reduction in the amount of transcripts of this gene.

**Figure 6:**
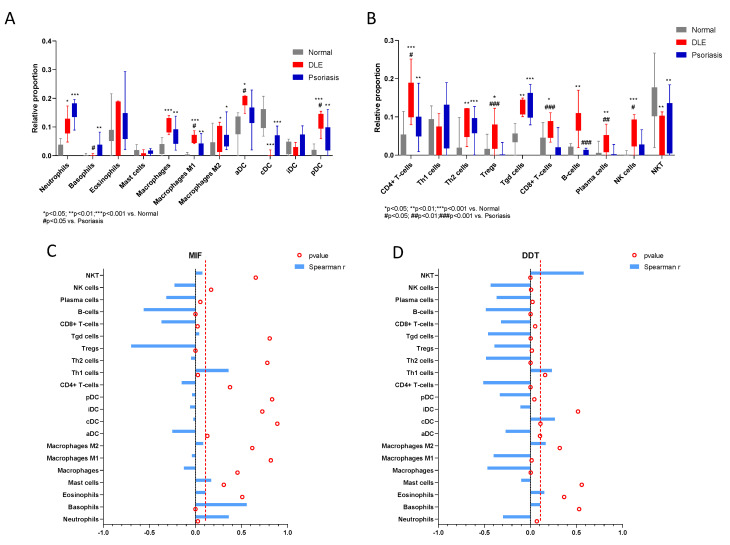
Correlation between MIF and DDT and infiltrating immune cells in DLE. The relative proportion of infiltrating immune cells in DLE skin samples, as compared to normal and psoriatic samples was performed using xCell (http://xcell.ucsf.edu/) software on the gene expression data obtained from the GSE52471 dataset. Data are provided separately for the myeloid cell populations (**A**) and the lymphoid cell populations (**B**). Differential analysis of immune cell abundance among the groups of samples was performed using the non-parametric Kruskal–Wallis test, followed by Dunn’s post hoc test. (**C**) Correlation analysis between MIF and infiltrating immune cell populations in DLE samples. (**D**) Correlation analysis between DDT and infiltrating immune cell populations in DLE samples. Correlation analysis was performed using the non-parametric Spearman’s test. The dashed line represents the threshold of significance, i.e., *p* < 0.05.

We also wanted to determine whether MIF and DDT levels correlated with specific infiltrating immune cell populations. To this aim, we first performed a deconvolution analysis on the GSE52471 dataset. As shown in Figure 6, DLE samples were characterized by significant higher infiltration of several populations of both the myeloid and lymphoid lineages (Figure 6). Interestingly, a diverging pattern of infiltration was observed between DLE and psoriasis samples for Tregs, cytotoxic T cells, and B cell/plasma cells (Figure 6B).

Correlation analysis revealed a positive correlation between MIF and neutrophil and basophil proportions, as well as Th1 cells, while a significant negative correlation was observed between MIF and Tregs, CD8+ T cells, and B cells (Figure 6C; Table 3). Similar data were observed for DDT, which showed a negative correlation also with M1, Th2, Tgd, and NK cells and a positive correlation with NKT cells (Figure 6D; Table 3).

## 3. Discussion

We have presently observed for the first time that reduced levels of MIF negatively correlates with the inflammatory status of DLE lesions. The in vivo data were strengthened by the bioinformatic analysis that also revealed a reduced expression of its homologue DDT in DLE lesions. The use of whole-genome expression data has been extensively used by ourselves and others for the identification of novel pathogenic pathways and therapeutic targets in several human pathologies including, autoimmune diseases [7,27,28] and cancer [26,29,30]. Despite the microarray dataset only showing a trend of reduced levels of MIF in DLE lesions as compared to the normal skin, entailing an adjusted *p* value = 0.18 (unadjusted *p* = 0.08), however, a significant downregulation of DDT could be observed in DLE as compared to healthy skin biopsies. Overall, the pattern of modulation of MIF, also in consideration of the concordant modulation of MF and DDT in other settings [6,25,26], suggests that the lower levels of MIF detected by immunohistochemistry are, at least partially, dependent on the lower amount of MIF mRNA in DLE. It is likely that the significance was not reached in the microarray because of the limited number of samples (seven DLE and 13 healthy controls), along with the variability in the expression levels of this gene.

We do not know whether the reduced expression of MIF in the presence of high level of inflammation may depend on a reduced transcription of this gene or, alternatively, on increased rate of degradation of the mRNA, that could be mediated by epigenetic phenomena, such as promoter hypermethylation, histone acetylation, or increased expression of specific miRNAs. Interestingly, the pattern of modulation of MIF in DLE appears to be strikingly different for psoriasis. Indeed, our microarray analysis revealed a significant increase in MIF (but not DDT) expression in psoriasis as compared to normal samples, which is in accordance with a previous report by Steinhoff et al. [31]. This is likely to reflect the different clinical and etiopathological features between DLE and psoriasis. Indeed, contrary to DLE lesions, psoriatic plaques do not lead to hair loss or epidermal atrophy [32], and antimalarial drugs may exacerbate the disease. Additionally, activation of the IFN-γ pathway is more predominant in DLE rather than psoriasis, which is characterized by a Th17-skewed signature [33].

This present finding is in contrast with the previously discussed pathogenetic role of MIF and DDT in multiple autoimmune diseases, including SLE, despite the possible occurrence of someoverlapping immune-pathogenetic features of this disease with DLE that are witnessed by the higher risk conferred from DLE to SLE development [4]. In a similar manner, MIF seems to play a pathogenetic role in immune-inflammatory and autoimmune diseases of the skin, including psoriasis, atopic dermatitis, eczema and UV radiation damage [34].

The reason of this apparent protective unicity of endogenous MIF and DDT in negatively correlating with inflammation in the skin in DLE remains to be studied, and development of in vitro and in vivo models of DLE will help to dismantle this point. It is also worth pointing out that MIF expression is significantly upregulated by growth factors, for instance transforming growth factor-β and platelet derived growth factor [35] and that MIF inhibits the tumor suppressor gene, p53 [36]. Hence, we could speculate that in overcoming p53 activity, MIF might stimulate epidermal cell growth and damage repair in DLE lesion. We are not currently able to determine whether the inverse correlation of MIF and inflammation represents an epiphenomenon, associated to the wound healing processes that follow the acute inflammatory phases, or whether MIF actually takes place in regulating inflammation in the skin. It is of interest in this setting that recent evidences indicate a key role of the MIF/CD74 cell signaling pathway in regulating wound healing after injury [37]. However, it should also be noticed that MIF is mainly produced byTh2 cells, rather that Th1 cells [38], hence, it may take a role in the resolution phase of the disease.

However, in agreement with emerging anti-inflammatory properties of MIF and DDT, we propose that these effects may be mediated by their abilities to activate of AMP kinase (AMPK), which is an endogenous regulator of inflammatory responses [39]. In a similar manner, potential beneficial effects of endogenous MIF have also been suggested to occur via inhibition of apoptosis, the induction of autophagy, regulation of homeostasis, and the recruitment of immunoregulatory cells [40].

This apparent protective effects of endogenous MIF and DDT in DLE adds this disease to the list of pathologies that may benefit from either endogenous or exogenous MIF (and DDT), as it has been shown in vitro and/or in vivo in models of amyotrophic lateral sclerosis [41,42,43], as well as in ischemic preconditioning-mediated cardio-protection [44] and the protection in acute kidney injury after cardiac surgery [45].

In addition, MIF appears to play a dichotomic role also in AD and PD [46,47], which may depend on multiple factors including phenotype of the patients and stage of the disease. 

Taken as a whole, these data warrant studies aimed at understanding the immunobiological basis responsible for the potential anti-inflammatory effects of MIF and DDT and the precise identification of genetic and clinical factors that may favor the emergence of the anti-inflammatory profile of these cytokines. In the context of DLE, our study opens diagnostic and therapeutic perspectives that may allow using local expression of MIF and DDT in cutaneous lesions of DLE patients as a biomarker. In addition, though we have presently not observed modification of MIF and DDT expression in response to treatment, it will be interesting to evaluate in larger cohorts of DLE patients whether or not the insufficient expression of MIF and DDT in the cutaneous lesions may be associated with a poorer therapeutic response. Studies of genetic polymorphisms of MIF in DLE seem also warranted.

From the pharmacological point of view, given the progressive development of specific MIF and DDT inhibitors in the clinical setting [8], it will be important to ascertain the safety of these agents in patients with DLE and also to consider the inclusion of ad hoc safety studies to rule out possible iatrogenic induction of DLE lesions. For example, patients with active DLE lesions should be careful in using immunomodulatory agents that may downregulate the production and/or function of MIF [8] including, for example, ibudilast approved in Japan for asthma, which is now being repurposed for immunoinflammatory diseases [48], as well as anti-MIF mAb that has completed phase I/II testing in cancer patients and the anti-CD74 mAb milatuzumab, which is approved for patients with multiple myeloma [49].

Along these lines, certain natural products, containing isothiocyanates, such as from broccoli, that are very potent inactivators of MIF tautomerase activity, should also be carefully consumed from patients with DLE [50].

Finally, the further demonstration of a protective role of MIF and DDT in the pathogenesis of DLE might offer novel therapeutic avenues for the treatment of this disease, which could consist of local or systemic application of specific agonists. 

## 4. Materials and Methods 

### 4.1. Patients

Thirty-seven patients with DLE were recruited for this study (Table 1). Biopsies were performed after a six-month wash-out period from topical treatments. The tissue samples were obtained by incisional skin biopsies, then fixed in 10% buffered formalin for 12 hours and then included in paraffin. Next, sections of 3–4 micrometers were stained in hematoxylin and eosin and evaluated histologically to confirm the diagnosis of DLE. The morphological diagnosis was made on the basis of established criteria [51]. We assessed the extent of the inflammatory infiltrate by staining with hematoxylin and eosin and quantified it according to a score (0; 1+; 2+; 3+). The study was approved by the local ethical committee of the University of Catania, Italy (n. 75/2020/PO).

### 4.2. Immunohistochemistry

Immunohistochemical analysis was performed as previously described [52]. After a standard pre-treatment the sections were incubated with anti-MIF polyclonal antibodies (PA5-27343 Thermofisher Scientific, Rodano, Italy), diluted 1:500 in PBS (Sigma, Milan, Italy). The immunoreaction was visualized by incubating the sections for 4 min in a 0.1% 3,3-diaminobenzidine (DAB) and 0.02% hydrogen peroxide solution (DAB substrate kit, Vector Laboratories, Burlingame, CA, USA). The sections were lightly counterstained with Gill’s hematoxylin (Histolab Products AB, Göteborg, Sweden) and mounted in GVA mountant (Zymed Laboratories, San Francisco, CA, USA). Immunohistochemistry-positive staining was defined as the presence of brown color.

### 4.3. Evaluation of Immunohistochemistry

Immunostained slides were separately evaluated by two pathologists, who were blinded to patient identity, clinical status and group identification, using a light microscope, at 200× magnification. The MIF-staining status was identified as either negative or positive. Immunohistochemistry positive staining was defined as the presence of brown chromogen detection within the cytoplasm. The percentage of MIF positive cells (Extent Score (ES)) was independently evaluated as the percentage of the final number of 100 cells, grouped in four categories: <5% (0); 5–33%(+); 33–66% (++); and >66% (+++). 

### 4.4. Microarray Selection and Analysis

In order to determine the expression levels of MIF in DLE patients and healthy controls, we searched the Gene Expression Omnibus database (https://www.ncbi.nlm.nih.gov/gds). For this study, the GSE52471 dataset was chosen, as it included whole genome expression profiles of skin biopsies from seven DLE patients, 13 healthy donors, and 18 psoriatic patients [33]. Up to now, this is the only dataset comprising both healthy donor samples and psoriatic samples along with DLE samples. Detailed information about the DLE patients is available in Jabbari et al. [33]. The Human Genome U133A 2.0 gene chip was used for the generation of this dataset. Raw data were preprocessed using the GCRMA (Guanine Cytosine Robust Multi-Array Analysis) algorithm and batch effect corrected using COMBAT. The submitter-supplied gene expression matrix was used for the generation of the data presented in the current study.

### 4.5. Statistical Analysis

Expression data are presented as mean ± standard deviation (SD). The LIMMA (Linear Models for Microarray Analysis) R package was used for identifying differentially expressed genes. An adjusted *p*-value < 0.05 was considered as threshold of significance. The Fisher’s chi-square test was used to compare the levels MIF expression in relation to the inflammatory score. In order to evaluate the proportions of the infiltrating immune cell subsets in DLE samples, as compared to healthy and psoriatic samples and to correlate them with the expression levels of MIF and DDT, we performed a computational deconvolution analysis. To this aim, we have used the web-based utility, xCell (http://xcell.ucsf.edu/) [53], a computational tool that is able, by using gene signatures, to infer the presence in a sample of various cell types, such as T helper cells, B lymphocytes, monocytes, and dendritic cells. Differential analysis of immune cell abundance among the groups of samples was performed using the non-parametric Kruskal–Wallis test, followed by Dunn’s post hoc test. Correlation analysis was performed using the non-parametric Spearman’s test. Unless otherwise indicated, a *p*-value < 0.05 was considered statistically significant. Statistical analyses were performed with GraphPad Prism 5 (GraphPad Software, San Diego, CA, USA) and SPSS 24 (IBM SPSS Statistics, IBM Corporation, Armonk, NY, USA).

## Figures and Tables

**Table 1:** DLE patient clinical data.

**Age (Mean ± S.D.)**		
		44.5 ± 14.8 years
**Sex**		
	Male	28.60%
	Female	71.40%
**BMI (Mean ± S.D.)**		27 ± 2.3
**Response**		
	Complete remission	42.80%
	Partial remission	14.30%
	Remission with hyperchromic/cicatricial results	42.90%

**Table 2:** Correlation between MIF and DLE clinical data.

MIF		AGE	SEX	BMI	RESPONSE
Spearman’s test	Correlation Cofficient	−0.272	0.036	0.202	0.610
	Sig. (2-taled)	0.233	0.877	0.380	0.792

**Table 3:** Correlation between MIF and DDT and infiltrating immune cells in DLE.

	MIF			DDT		
	Spearman R	95% Confidence Interval	P (Two-Tailed)	Spearman R	95% Confidence Interval	*p* (Two-Tailed)
Neutrophils	0.3605	0.03638 to 0.6160	0.0262	−0.2972	−0.5700 to 0.03468	0.07
Basophils	0.557	0.2797 to 0.7485	0.0003	0.1053	−0.2312 to 0.4192	0.5294
Eosinophils	0.1106	−0.2260 to 0.4237	0.5084	0.1508	−0.1869 to 0.4566	0.3662
Mast cells	0.1699	−0.1679 to 0.4720	0.3077	−0.0983	−0.4134 to 0.2378	0.5571
Macrophages	−0.1244	−0.4351 to 0.2127	0.4567	−0.4653	−0.6885 to −0.1615	0.0032
M1	−0.03855	−0.3624 to 0.2936	0.8183	-0.3985	−0.6428 to −0.08064	0.0132
M2	0.08375	−0.2516 to 0.4012	0.6172	0.1662	−0.1717 to 0.4690	0.3187
aDC	−0.2505	−0.5349 to 0.08497	0.1293	−0.2675	−0.5478 to 0.06682	0.1045
cDC	−0.02356	−0.3493 to 0.3073	0.8883	0.2643	−0.07027 to 0.5454	0.1089
iDC	−0.05881	−0.3799 to 0.2749	0.7258	−0.1085	−0.4219 to 0.2280	0.5166
pDC	−0.03558	−0.3598 to 0.2963	0.8321	−0.3312	−0.5949 to −0.003070	0.0422
CD4+ T-cells	−0.148	−0.4544 to 0.1897	0.3752	−0.5121	−0.7195 to −0.2208	0.001
Th1 cells	0.3598	0.03551 to 0.6155	0.0265	0.2329	−0.1035 to 0.5214	0.1594
Th2 cells	−0.04677	−0.3695 to 0.2861	0.7804	−0.4816	−0.6994 to −0.1819	0.0022
Tregs	−0.698	−0.8350 to −0.4795	<0.0001	−0.3901	−0.6369 to −0.07068	0.0155
Tgd cells	0.04125	−0.2911 to 0.3648	0.8057	−0.459	−0.6843 to −0.1537	0.0037
CD8+ T-cells	−0.3673	−0.6208 to −0.04415	0.0233	−0.3184	−0.5856 to 0.01120	0.0514
B-cells	−0.5602	−0.7506 to −0.2841	0.0003	−0.4842	−0.7012 to −0.1852	0.0021
Plasma cells	−0.316	−0.5838 to 0.01392	0.0533	−0.3668	−0.6205 to −0.04356	0.0235
NK cells	−0.2272	−0.5170 to 0.1095	0.1702	−0.4307	−0.6650 to −0.1190	0.007
NKT	0.07496	−0.2599 to 0.3937	0.6547	0.577	0.3067 to 0.7612	0.0001

## Data Availability

The data presented in this study are available on request from the corresponding author.
